# Kenyan Nurses Involvement in National Policy Development Processes

**DOI:** 10.1155/2014/236573

**Published:** 2014-10-02

**Authors:** Pamela Atieno Juma, Nancy Edwards, Denise Spitzer

**Affiliations:** ^1^Nursing Department, Aga Khan University, P.O. Box 39340, Nairobi 00623, Kenya; ^2^African Population and Health Research Center, P.O. Box 10787, Nairobi 00100, Kenya; ^3^School of Nursing, University of Ottawa, 1 Stewart Street, Ottawa, ON, Canada K1N 6N5; ^4^Institute of Feminist and Gender Studies, Faculty of Social Sciences, University of Ottawa, 1 Stewart Street, Ottawa, ON, Canada K1N 6N5

## Abstract

The aim of this study was to critically examine how nurses have been involved in national policy processes in the Kenyan health sector. The paper reports qualitative results from a larger mixed method study. National nonnursing decision-makers and nurse leaders, and provincial managers as well as frontline nurse managers from two Kenyan districts were purposefully selected for interviews. Interviews dealt with nurses' involvement in national policy processes, factors hindering nurses' engagement in policy processes, and ways to enhance nurses' involvement in policy processes. Critical theory and feminist perspectives guided the study process. Content analysis of data was conducted. Findings revealed that nurses' involvement in policy processes in Kenya was limited. Only a few nurse leaders were involved in national policy committees as a result of their positions in the sector. Critical analysis of the findings revealed that hierarchies and structural factors as well as nursing professional issues were the primary barriers constraining nurses' involvement in policy processes. Thus, there is need to address these factors both by nurses themselves and by nonnursing decision makers, in order to enhance nurses engagement in policy making and further the contribution to quality of services to the communities.

## 1. Introduction

With the global debates around health policy reforms, there have been increasing calls for nurses to be actively engaged in national policy processes [1–3]. This engagement has been encouraged for two main reasons. First, nurses comprise the majority of the global health care workforce, working closely with patients and their families in a variety of settings. Thus, nurses' experiences and insights may help guide improvements in the quality of health service delivery and inform health systems strengthening. Second, many health sector policies have an impact on nurses' work environments and consequently their professional practice. Thus, nurses' input on health sector policies could help ensure that supportive work environments for clinical practice are taken into account when policies are reformed. Given nurses' pivotal role in health care delivery, their engagement in policy changes in the system has been described as both a moral and professional obligation as well as a responsibility to the people they serve [2, 4]. The engagement of nurses in national policy processes is especially important in lower income settings where nurses comprise an even larger proportion of the health sector workforce and where the largest burden of illness exists.

Historically, nurses have had limited involvement in national policy and political decisions that affect health service delivery [5–8]. For instance, a quantitative study of national nurse leaders and hospital based nurses in Thailand revealed that majority of nurses were not involved in policy formulation or modification stage. Rather they were involved in policy implementation. Literature further reveals several reasons for nurses underengagement in policy including lack of awareness of policy related issues and processes, inadequate knowledege and skills on how to contribute to policies, and lack of opportunities to contribute to policy decisions [9–11]. Nurses' limited participation in policy processes has also been attributed to gender differences in the health care system [12, 13]. Nursing is predominately viewed as a woman's occupation involving caring for others, while men are typically seen in positions of power and decision-making [14–16]. Thus, men are more likely than women to be involved in public policy and to influence policy decisions. In Kenya, nurses' underengament has also been attributed, in part, to an inadequate policy focus in undergraduate and graduate nursing programs [16].

Studies provide several strategies that can be applied to enhance nurses' engagement in policy processes. Several studies have emphasized that nurses can engage in policy processes directly by assuming formal leadership positions in the health care system, and indirectly through lobbying and advocacy with policy-makers via professional organizations [12, 17]. The nurse leaders can identify issues strategically, work with decision-makers, and understand who holds the power in the health care organizations and who controls the resources for health care in order to influence them [18]. Another strategy is to build nurses' understanding of and competencies in policy and politics by integrating pertinent courses in nursing training and fellowship programs [17–20]. Examples of programs developed to enhance nurses' competency in leadership and policy influence exist mainly in developed countries [21, 22]. For instance In the USA a program was developed to train nurse leaders who would influence policy change [17]. The training helped the nurse leaders to understand how policy reforms affect health care, the policy formulation process, and the challenges affecting health care including access, quality, and care as well as the role of media.

Several authors have emphasized the need for nurses to utilize research as credible evidence to inform policy decisions. However, the studies have found that nurses have not been adequately supported to generate evidence to inform health policies [23–26]. Furthermore, nurses from low- and middle-income countries (LMICs) have inadequate training and mentoring in research as well as scarce resources for conducting research [27]. Thus, there is a need to strengthen LMIC nurses' research abilities to enhance their ability to understand, generate, and utilize research knowledge that is useful for policy change.

Very few emperical studies have examined extent of nurses' involment policy developemnt process in the African context. A survey of nurse leaders involvement in health policy process in East Africa found that nurse leaders' participation in all stages of policy development was limited and inconsistent [28]. Another study looked at nurses involvement in HIV/AIDS policy in four LMICs including Kenya and Uganda [29]. The study revealed nurses' lack of involvement in policy processes, poor communication of policies from the national level, and a lack of resources for nurses to implement the policies. Published studies are mainly descriptive reviews and have not used a critical theory lens to analyze nurses' contributions to policy processes. Furthermore, studies have rarely compared the perspectives of nurses and other decision-makers regarding nurses' engagement in policy development and implementation processes. This study applied a critical theory perspective to examine how nurses had been involved in national health policy processes in Kenya, eliciting the perspectives of nurses and decision-makers working at various levels of the health care system.

## 2. Theoretical Underpinnings

This study was guided by critical and feminist theories. Critical theory aims to examine hierarchies, power and domination inherent in policy processes [30]. These hierarchies may influence how people participate in policy decision-making. Feminist theories are related to critical theory but focus on gender domination and discrimination within patriarchal societies [28, 29]. From feminist perspectives, analysis examines formal power structures that create or maintain gender inequality, thereby systematically elevating men above women [31, 32]. In this study, critical theory helped interrogate historical hierarchies and structures as well as gender-related issues affecting nurses' participation in policy processes.

## 3. Methodology

The overall study applied a two-phased mixed method (qualitative/quantitative) design to examine the influence of policies on nurses' work as well as how nurses were involved in policy processes in the Kenyan health care system. This paper focuses on the qualitative results, describing how nurses were involved in national policy processes in the Kenyan public health sector. The perspectives of nonnursing decision-makers, national level nurse leaders, and frontline nurses and managers were sought. The specific objectives were (1) to analyze the extent of nurses' involvement in the national policy development processes in the health sector; (2) to analyse the reasons for nurses' inadequate involvement in the national policy development in the health sector; and (3) to describe mechanisms though which nurses' involvement in policy development processes can be improved.

### 3.1. Study Setting

The study was carried out in the Kenyan public health care system, since this sector has primary responsibility for policy development and provides about 55% of health care services [33]. The health delivery system is organized in a pyramidal structure with six levels of care. The community is the first level, followed by dispensaries and medical clinics, health centers and nursing homes, district level hospitals, and provincial level and national level hospitals [34]. Decision-making structures exist at national, provincial, and district levels, all of which include nurse representation.

Nurses comprise 45.3% of the public health sector workforce in Kenya [16]. Although these nurses work at all levels of the health care system, the majority are employed at the district levels [16]. The majority of nurses entered the health care service with diploma or certificate level preparation until the early 1990s when the country started training degree level nurses. Kenya's nursing data base for 2011 indicates that among those employed in the public sector, the majority were certificate nurses (53%), followed by diploma nurses (46%), with a small minority of degree level nurses (1%) [34].

### 3.2. Sampling

Decision-makers at national, provincial, and district levels were purposively sampled based on their positions involving policy development or implementation. National level nonnursing and nursing decision-makers employed by the Ministry of Health were eligible for selection. Two provinces (Nyanza and Central) were purposively selected because of their varied geographical locations and differences in health indicators. Nyanza province had poor health indicators and few health service resources, while the Central province had better health indicators and more health service resources [35]. One district was then purposefully selected from each of these provinces and the two main district public hospitals were the sites for sampling district-level nurses and managers. Nurses and nurse managers in these district hospitals were selected based on a sufficiently long work history in the system and 2 years or more in the facility which gave them potential familiarity with many of the policy changes affecting nursing and health system reforms in Kenya.

### 3.3. Data Collection

Open-ended interview guides tailored to participant groups were used with all key informant interviewees. The groups included national level nursing and nonnursing decision-makers, decision-makers at provincial and district levels, and frontline nurses and managers at the hospital level. Participants were asked questions about national level health policy-making processes; extent to which nurses engaged in policy-making and implementation processes; and ways to improve nurses' involvement in policy processes. Data collection was stopped when saturation was reached. Additionally, the researcher kept field notes during the visits. Interviews took 45 to 60 minutes and were audio taped with participants' consent.

### 3.4. Data Analysis

Audio-taped data were transcribed verbatim and they were analyzed simultaneously with data collection. Transcripts from each participant were read repeatedly before and after coding to gain a sense of the whole interview. Interview data were clustered in three groups including nonnursing decision-makers, nurse leaders and frontline nurses, and nurse mangers; then all the data sets and field notes were triangulated as the analysis proceeded. Content analysis of data was carried out. Data was initially coded and divided into contents including nurses' engagement in policy, reasons for under engagement, and ways to improve nurse' engagement. Matrices were then used to compare categorized responses of national level nonnursing decision-makers, national nurse leaders, and frontline nurses and managers [36, 37]. Similarities and differences in the participants' responses were also compared by these groupings. Further analysis and interpretation of findings was then done using critical and feminist lens to identify hierarchies and gender related factors that influenced nurses' engagement in policy development.

### 3.5. Rigor

Credibility was enhanced throughout the study process by maintaining accuracy in data collection, analyzing, and reporting. Audiotapes and verbatim transcription preserved the accuracy of the data. Critical reflexivity involving cyclical and continuous comparisons of data collected were used throughout data collection and analysis. These included journaling and writing reflections on processes and decisions [36, 37]. Transferability was enhanced through the purposive recruitment of participants at different health care system levels. These participants had a range of experiences with national policies. Triangulation of data from different types of respondents was done making it possible to compare and confirm similar perceptions across different groups.

### 3.6. Ethical Approval

Ethical approval was obtained from University of Ottawa Health Sciences and Science Research Ethics Board in Canada and Great Lakes University of Kenya Ethics Review Committee. Administrative approval was also obtained from the Ministry of Health in Kenya, and appropriate authorities in provincial health offices, district offices, and the two district hospitals where study participants were recruited. Informed consent was obtained from each participant. Participants' privacy was maintained during interviews by interviewing them in a private room. No personal identifying information was included in the transcripts.

## 4. Findings

A total of 32 interviews were conducted (Table 1). At the national level, five interviews were conducted with nonnursing decision-makers at the Ministry of Health, one interview with the World Health Organization country representative and three interviews with national nurse leaders. Each of the three national nurse leaders had risen up the ranks from the district level to the national level and had worked for more than 20 years in public service. At the provincial level, two nonnursing decision-makers and two nurse leaders were interviewed in Nyanza and one nonnursing decision-maker and two nurse leaders interviewed in Central province. At the district level, one nurse manager in each district office and seven frontline nurses and managers in each district hospital were interviewed.

### 4.1. Nurses' Involvement in National Policy Processes

Most respondents were of the opinion that nurses were not adequately involved in the health sector policy development processes at the national level. They mentioned that only three nurse leaders from the offices of the Chief Nurse, the Nursing Council and the, National Nurses Association had been invited to participate in national policy committees as part of their jobs. The nurse leaders' input on these committees was described as revolving around representing nurses' issues at selected policy tables, communicating policies to nurses, and regulating nursing education and practice. The Chief Nurse was charged with the highest responsibility of representing all Kenyan nurses at policy meetings. The other two nurse leaders indicated that they attended fewer meetings. Two of the nonnursing decision-makers felt that Kenyan nurses were adequately involved in national policy-making processes through the nurse leaders' representation at the national level. In contrast with this view, all nursing participants felt that this representation was inadequate given the large number and diversity of nurses in the country.

Generally nurses have not been fully involved in policy formulation. I believe nurses need to know the reason behind the formulation of specific policies. Many a time nurses are involved after policies have been formulated, and maybe just a few nurses [national nurses leaders are involved in the policy formulation stage]; the province is just informed later that this is happening, but really there has been no good input from nurses. (National nurse leader)

Furthermore, the contributions of these three nursing leaders to the national policy formulation processes were considered inadequate by both the nurse leaders and nonnursing decision-makers but for different reasons. While nonnursing decision-makers emphasized the limitation of nurse leaders' singular focus on nursing issues, the nurse leaders described the problem of inadequate representation of nurses on a wider set of national policy committees. They described representation on these committees as being primarily heads of departments, the majority of whom were doctors. The Public Health Act, which allows doctors to be the heads of ministerial departments, as well as provincial and district health offices, affords doctors this role.

Normally the heads of the various departments sit down; many of them are headed by doctors because of the Public Health Act, and others would be the technical people. Nurses and other health officers are not well represented. (National nurse leader)

The findings also revealed nurse leaders' uneven participation in policy meetings. According to the nurse leaders, nurses only attended those policy meetings requiring reports on nurse situations, while doctors were active in every policy meeting regardless of the policy content. Furthermore, nurse leaders noted that many of the final policy decisions had been made without nurse leaders being present.

Frontline nurses and nurse managers at district levels described their primary policy role as being implementers of national policies. Even though policy-making was considered a national level activity, nurses at the frontline were concerned that they were not involved. They added that their contribution could be important since nurses better understood the care environment.

Nurses should be consulted before policies are started because we are the majority, but they decide for us (…). Nurses have everything that they need,… education, brains, but they lack chance to air views. They stamp on what is already decided. We should decide and give the feedback at national level from lower level. (Provincial nurse leader)

A common view, among frontline nurses, was that they were excluded from policy decisions even in the workplace.

We are not involved, yet we are the ones who really are on the ground, we are the backbone of the hospitals. The other people come to write papers and go away (…). We are like a mother in the kitchen, we are the ones who know how much we need, so we should be really involved in every step, because you cannot just tell me “now cook” and even I was not there in the shopping but you want to see what I am cooking; we should be involved from the word go. (Frontline nurse)

Due to their limited involvement, frontline nurses and managers felt that their priority work-related issues such as staff shortages, motivation, and resource inadequacies were not being addressed. Yet, they were expected to deliver quality services. They felt that policies were just pushed on them, so they sat back and watched or did only what they could, as one nurse manager commented:

Lack of nurses' involvement in formulation is the cause of poor policy implementation. Policies are pushed on them, owning them becomes a challenge. They are not positive towards it, but since they have to comply, they do it, but not in the right way. When you are involved from the beginning, you understand and own it, and during implementation, you have inward drive to accomplish what you planned (…). We feel like we are pushed at the corner, so we remain there and watch things as they are done, or do things because they must be done, not that we feel motivated to do them.

### 4.2. Reasons for Inadequate Involvement of Nurses

The participants gave several reasons as to why there was inadequate nurses' participation in national health policy processes. One reason was that policy-making primarily followed a top down approach, emanating from the national level. Respondents felt that this approach denied nurses opportunities for direct involvement in national policy decisions because most nurses are employed at lower levels of the health care system. Nurses viewed policies as prescriptions from above that they were supposed to follow. As one nurse manager said “*for me policy-making is a thing for those at the top, whether they are doing the right thing or not, to them it does not matter.”* Another frontline nurse commented that “*policies are made from above, they are not made according to how you want things to be done, they are just brought and you are supposed to follow them.”*

Other reasons for nurses' lack of engagement in policy formulation mentioned by nonnursing decision-makers were their lack of knowledge about the policy-making process and lack of skills to engage in this process. Nurse leaders concurred that nurses did not generally understand the broader issues outside nursing that were necessary to influence policies. They largely blamed this on the omission of any curriculum related to policy issues in basic nursing training. Others felt that nurses' low participation was due to the general design of the health care system, which excluded nurses from policy-making structures.

It is by design of health system that nurses are not brought at the policy tables… [The] majority of nurses lack knowledge and skills (…) to support that policy. The thing is to bring people who would add value at the table. Therefore nurses should be made to add value when an opportunity comes for them to sit at the policy-making tables, not to be a passenger but to be a participant. (National nurse leader)

Nurses were also perceived as being isolated from other professionals. Nonnursing decision-makers blamed the nursing profession for its tradition of being a “closed” professional group, concerning themselves only with professional issues and not broader issues affecting the health care system. A few of the decision-makers felt that nurses had failed to market themselves as important providers in the health care system. In addition, they felt that nursing had a hierarchical system that hindered junior nurses from participation. One national nonnursing decision-maker was particularly strident in his/her remarks:

Nurses are allergic to change. They behave like the military in uniform, taking orders. Nursing profession discourages nurses from thinking because it's the matron who decides, (…). They have put a self-imposed mind block by relying on another person to make decisions (…). They go through a very hierarchical system unlike other professions. While a nurse will still report to a nursing officer, she cannot make decisions on her own, it is the biggest weakness, there is no freedom, and they maintain the status quo. (Nonnursing national decision-maker)

Another reason expressed for nurses' nonengagement was the inability of nurses to bring forward research evidence to inform policy formulation. Nurse leaders commented that nurses had inadequate skills for generating and utilizing research evidence to influence policy decisions at national levels. Frontline nurses complained that a further impediment was the failure of nurses to take united action to influence policy. They had different professional associations (the Kenya Registered Nurses' Association and Kenya Professional Nurses Association), which served different nursing groups and tended to divide nurses and their leaders.

### 4.3. Enhancing Nurse Participation in Policies

Participants gave several suggestions regarding how nurses' participation in the policy process could be enhanced. They mentioned actions to be taken by decision-makers to enhance nurses' participation in policy-making. These included creating more opportunities for nurses to contribute to policy processes, increasing nurses' representation on policy-related structures, and reviewing the public health law to allow nurses to compete for leadership positions in the system. In addition, frontline nurses felt that national policy-makers should consult frontline nurses when developing national policies. It was their view that this would enhance bottom-up input on policy-making. As one frontline nurse said:

I believe there is a way of channeling suggestions until they reach the national level, maybe through the nurses in charge of hospitals to the provincial then to the national level through writing and meetings.

As their representatives, the frontline nurses felt that nurse leaders should come to the ground more often and get views from frontline nurses to include in policies:

Nurse leaders are at the top, when there are issues to be discussed, they can come down, involve us in discussions and gather information, so that when they make these policies they know it is something that has been discussed, it has been understood and it is needed, because occasionally policies have been made [and] when they come down here they do not even work. (Frontline nurse manager)

Some nursing participants mentioned that the opinion of frontline nurses could be sought through research and regular meetings and then brought to policy-making processes.

Participants also described actions to be taken by the nursing profession. In particular, they identified the need to empower nurses through training to enable them to better understand and participate in policy formulation. While the experiences of nurses were thought to be important assets for this input, a broader knowledge of the health care system, of policy-making, and of decision-making processes, were described as essential prerequisites for constructive contributions.

The bottom-line is that you must be knowledgeable, you may be a real nursing guru, (…) your contribution should not only be based on care, of course care is very critical, but you must have general knowledge like how the government operates (…). Majority of nurses do not know anything apart from nursing, you are supposed to have general knowledge, like International Monetary Funds policies, how does it affect us?, what happened? [sic]. (Nurse leader 2)

Other areas identified for skill-building included political skills needed to engage the government to address policy gaps that affected nurses' work as one nurse leader said that* “nurses in this country lack political skills, they should have political skills in order to dialogue with politicians and other leaders in this country*.” In addition the participants mentioned that nurses need data management and research skills to bring evidence to policy. They mentioned that nurses could bring evidence to policy meetings and conferences.

Finally, they mentioned actions to be taken by nurses themselves to enhance their ability to participate in policy-making. Nonnursing decision-makers strongly suggested that nurses should take the lead to change their situation. They mentioned that nurses should market themselves, progress in their careers, diversify, venture outside the nursing profession, and broaden their curriculum in order to understand broader policy related issues.

Nurses build empires around themselves (…). Nurses must be free to venture into other areas and be competent in those areas and not tied to nursing. The ball is in nurses' court. We are ready to embrace and support (…). The medical profession is much more marketized than nursing. More often, you find doctors in areas, which are not related to medicine and they fit well. They do not have to link strongly to their profession, they are more independent. (National nonnurse decision-maker)

Congruent with this decision-maker's opinion, some of the nurse leaders interviewed added that more nurses should position themselves to take up leadership roles in the system. They indicated that nurses should diversify their knowledge, be united to build a strong voice, collaborate and network within and beyond the country, and be assertive about what they wanted. They added that the nursing council and the professional association should be strengthened to enhance their respective roles in influencing national policies that affect nurses' work.

Frontline nurses added that nurse leaders should be more proactive in policy activities so as to influence policy-makers. They called for nurse leaders to advocate for better work conditions for nurses. Some frontline nurses felt that this type of advocacy would be an incentive for front line nurses to participate in decision-making in the health care sector.

## 5. Discussion

From a critical perspective, two major issues influencing nurses' involvement in policy processes emerged. These issues were hierarchies and structures influencing decision-making in the health sector and nurse and nursing professional issues. These issues are discussed below.

### 5.1. Hierarchies and Structures

Three intersecting hierarchies hindered nurses' active engagement in policy processes in Kenya: health systems hierarchies, interprofessional hierarchies, and intraprofessional hierarchies. The Kenyan health systems decision-making structure is organized following political administrative levels including central headquarters and provincial and district levels. The political structures mandate the central administration as the policy-making and service planning level. The Kenyan health policy framework of 1994–2010 emphasized that policy formulation was the role of the central office [38]. This top-down policy-making process limited nurses' opportunities to engage directly in policy formulation in the health care system. This policy framework delineates a policy implementation rather than a policy formulation role for nurses. Given the top-down nature of policy-making processes in the health sector, particularly with the many stakeholders and foreign donors involved, the findings speak to the importance of finding ways to give nurses some voice in decision-making at all levels.

The interprofessional hierarchy described within the health sector identified medical doctors as being the dominant policy decision-makers at various levels of the health care system. Social constructs of racialized status and gender imbue health care hierarchies and are grounded in the legacy of Kenya's colonial period that ended in 1963 [16]. White colonialists developed health care institutions that favored a male- and white-dominated medical profession over a female- and African-dominated nursing profession. The education system prepared physicians with first degrees while nurses acquired certificates and later diplomas. Although baccalaureate degree nursing education in Kenya began in the late 1990s, only one percent of nurses in the public health care sector have baccalaureate degrees. Resultantly, medical doctors have higher status and are granted more decision-making authority in the system as opposed to nurses whose education and status were, and are considered low, further precluding nurses from higher decision-making positions.

The interprofessional hierarchy is further sustained by the legal system, specifically the Public Health Act, which led to the systematic exclusion of nurses from senior leadership positions at various levels of the health care system. According to the Act, physicians were to serve as the heads of various departments, administrative directors of districts and hospitals, and managers of various programs, while senior nurses could only be placed in positions to represent nurses, thus limiting their participation in policy and other health care decisions. This legislation thus legitimized the gendered professional hierarchy and further limited the types of issues that nurse leaders could bring to the policy table. Nurse leaders' participation at policy tables was considered* nursing centric *by both nursing and nonnursing leaders. While the majority of decision-makers blamed nurse leaders for nursing centric participation, this kind of participation was also shaped by the system, which mandated nurse leaders to report only on nursing issues. This is consistent with a global study [39] of nurse leaders focusing on Chief Nursing Officers, which revealed that, even though these nursing leaders had varied responsibilities associated with their positions, they were only asked to provide nursing-related advice.

The study revealed how an intraprofessional hierarchy within the nursing profession played out in the policy arena. Nurse leaders at the national level were recognized as the sole professional representatives of other nurses in the country who would provide input on national policy decisions. The intraprofessional hierarchy influenced channels of policy communication to frontline nurses who received new policy directives from their supervisors at every level of the system. This hierarchical pattern of decision-making within nursing may have been exacerbated by the fact that there are only a few nurses in key national level positions, limiting the scope of their policy influence at the national level and precluding them from engaging nurses more proactively at the district level or on the front lines in policy-oriented discussion. In addition, nurses at lower levels felt oppressed by senior nurses who made most of the decisions. From the findings, the nonnursing decision-makers had more to say about nurses' oppression than nurses themselves. Nonnursing decision-makers indicated that nursing was a profession where the oppression of lower level nurses was a problem. However, they were generally pessimistic, indicating that nurses were not yet ready to make a change. The oppression of lower cadre nurses by higher cadre nurses in decision-making has been documented in other studies [40–42]. In the absence of feedback from the grassroots on policy implementation, as identified by participants in this study, the voices of nurses at lower levels might be suppressed further. Given the fact that Kenya has a decentralized health care system, more participatory approaches could be introduced to enhance nurses' potential engagement in policy-making, thus improving bottom up planning and decision-making. This would increase the way in which those who have less power in a hierarchy get voice.

### 5.2. Nurses and Nursing Profession

Both nursing and nonnursing participants attributed nurses' underengagement in policy to issues related to the nurses and the nursing profession. Respondents consistently indicated that nurses had inadequate knowledge and skills required to contribute to policy processes. They described this deficiency in nurses' knowledge as being attributable to a failure of the nursing profession to incorporate policy issues and policy process into nurses' training curricula, thus maintaining the status quo for nurses' role in decision-making. Studies in other contexts have revealed how lack of policy content in nursing training contributes to nurses' lack of understanding of policy [43]. Given the need to strengthen nursing leadership to influence policies, other programs have emerged to train nurse leaders in ways to influence policy. In the African context, many nurse leaders have been enrolled and trained in a leadership training program offered by International Council of Nurses [16]. This participation may help strengthen nurses' competency in engaging in national health care decision-making.

Study findings also revealed that nurses had inadequate advocacy, research, and political skills required to influence policies. These issues are similar to what has been identified in studies from higher income countries [12, 17, 23]. In these studies, the profession is seen to have been slow in incorporating these issues in nursing education. Nonnursing decision-makers in this Kenyan study felt that the profession had reinforced its own weaker professional status by failing to enhance nurses' abilities and competencies to influence decisions at strategic levels in the system. Their comments reflected an openness to incorporate nurses' views if nurses were competent in national policy processes and more knowledgeable about general health care system issues.

The findings also revealed that nurses have worked in isolation from other professionals in the system. With the emphasis on professional transformation towards equality and diversity [44, 45] nurses cannot continue to build walls around themselves. This would require nursing champions to take up leadership in working with other health cadres to influence policy change. While leadership competencies have been recognized as key attributes to shaping and influencing policy [46, 47], in this study nursing leadership was viewed as internal to the nursing profession, with concerns about the focus on nurses' work issues and not general health care issues. Nursing champions should not focus on the profession alone; they must be educated in the broader health systems issues and become patient advocates for health care as they engage with other professionals in leading policy change [48]. This engagement would contribute to health systems improvement.

## 6. Study Limitation

We only collected data from Kenyan context. Therefore, the findings are only applicable to Kenya. Kenya has many district level hospitals but data from frontline nurses was mainly collected from two districts given the limitation in funding.

## 7. Conclusion and Recommendations

In conclusion nurses in Kenya have not been adequately involved in national policy development. Applying critical theory perspective in the analysis has revealed important health system structures and hierarchy as well as nursing professional factors hindering nurses' participation in policies making processes. These factors must be addressed if nurses' input in policy-making is going to be significantly altered. To enhance nurses' participation in policy processes, the policy-makers should address hierarchies, domination, and gender issues inherent in the health care system and establish mechanisms through which the views of nurses could get to national level decision-makers when policies are being formulated. This can be achieved by enacting legislation in Kenya that would allow nurses with competencies in leadership and policy to hold senior positions in the health care system just like doctors. With governance opportunities created through decentralization of power, there is a need for all health professionals to have equal opportunities to be in leadership and management positions that are embedded within the decision-making structures in the health care system. To enhance bottom-up policy-making, national level decision-makers should solicit the views of nurses at the frontline with regard to policies that might affect their work and quality of care. There are well-described processes for this purpose [49, 50] but they cannot be implemented in isolation of process changes at the district and national levels, which build mechanisms for continuous quality improvement.

Leadership and advocacy skills should be developed within nursing to enhance nurses' abilities to carry out policy advocacy at all levels. ICN has developed programs aimed at building the capacity for such leadership in societies around the world [51]. These programs include the Leadership in negotiation program, the global nursing leadership institute, and the leadership for change program. Nurses in this region can benefit from these programs. To enhance their role in advocacy, nurses should unite to build a strong voice to advocate for improvements in national policies that affect their work and quality of care. Working in collaboration with other health professionals, nurses can influence decisions to strengthen the health care system, resource allocation, and primary health care as well as patient care.

In addition, nursing regulators and educators should be clear that policy involvement is part of nurses' scope of practice. Nurses, particularly at the frontline, should be given orientation on existing national policies in the sector and how these policies are affecting their work environment and patient outcomes. This would be an important step towards better communication of policy impact, creating a much needed feedback chain between those implementing policy and those formulating policy. As members of nursing associations, frontline nurses should encourage and support their nurse leaders to speak for nurses at the national policy tables. In their workplaces, decision-makers should allow other nurses to actively engage in organizational policy decisions during policy implementation.

The nursing profession should consider and implement strategies to strengthen nurses' capacity to contribute to policy decisions that would enhance service delivery. In order to effectively do this, nurses in Kenya will need to understand that the policy cycle is complex and influencing policy decisions depends on having a sufficient understanding of the broader issues in the health care system, as well as knowledge and competencies in policy formulation, implementation, and evaluation. To enhance nurses' awareness, literacy, and competency in policy, nursing educators should introduce a course on policy and policy processes, as well as policy influencing in the nursing training curriculum.

Finally, the nursing profession should carry forward the notion of evidence-based policy-making. They should develop skills in research and conduct both health systems and clinical research to inform policy-making decisions. Research courses should be linked to policy courses and training curricula should include current and locally relevant examples of how evidence can be used to shape policies. This would include strategies and tools for communicating research to policy-makers, like writing policy briefs and position statements based on research evidence. Sharing of policy relevant research would contribute to policy change and resource allocation to improve nurses' work.

## 8. Further Research

This is one of the first studies examining the perspectives of both nurses and nonnurses at different system levels regarding nurses' involvement in national policy-making processes. With the continuing health care policy reforms, further examination of issues of hierarchy, gender, and power inherent in the health care system should be conducted in a broader context in Africa. The question is how do we unravel the deeply embedded hierarchies and power processes that sustain nurses' status quo in policy change process? Of particular importance is how the hierarchies affect the role and performance of nurses at lower levels and how can nurses influence national leaders to enhance participation in health policy change processes. Furthermore, this study did not carry out an in-depth examination of the competencies held by nurse leaders that would be useful for policy change; thus future research should examine these aspects.

## Figures and Tables

**Table 1:** Study participants.

Levels	Nonnursing decision makers		Nurse leaders		Front line nurses and managers		Total
	M	F	M	F	M	F	
National	4	2	1	2	—	—	9
Provincial					—		
Nyanza	2	—	1	1			4
Central	1		1	1		—	3
District	—						
Nyanza		—	—		4	4	8
Central			—		3	5	8

Total	7	2	3	4	9	7	32
